# Translation and validation of the German version of the Pet-Related Stress Scale

**DOI:** 10.3389/fvets.2025.1592569

**Published:** 2025-05-09

**Authors:** André Hajek, Ariana Neumann, Larissa Zwar, Razak M. Gyasi, Dong Keon Yon, Supa Pengpid, Karl Peltzer, Rosalie Corona, Shelby Elaine McDonald, Hans-Helmut König

**Affiliations:** ^1^Department of Health Economics and Health Services Research, University Medical Center Hamburg-Eppendorf, Hamburg Center for Health Economics, Hamburg, Germany; ^2^Department of Psychology, Brock University, St. Catharines, ON, Canada; ^3^African Population and Health Research Center, Nairobi, Kenya; ^4^Faculty of Health, National Centre for Naturopathic Medicine, Southern Cross University, Lismore, NSW, Australia; ^5^Center for Digital Health, Medical Science Research Institute, Kyung Hee University College of Medicine, Seoul, Republic of Korea; ^6^Department of Health Education and Behavioral Sciences, Faculty of Public Health, Mahidol University, Bangkok, Thailand; ^7^Department of Public Health, Sefako Makgatho Health Sciences University, Pretoria, South Africa; ^8^Department of Healthcare Administration, College of Medical and Health Science, Asia University, Taichung, Taiwan; ^9^Department of Psychology, University of the Free State, Bloemfontein, South Africa; ^10^Department of Psychology, College of Medical and Health Science, Asia University, Taichung, Taiwan; ^11^Department of Psychology, Virginia Commonwealth University, Richmond, VA, United States; ^12^School of Social Work, Colorado State University, Fort Collins, CO, United States

**Keywords:** pet-related stress, pet ownership, anxiety, depression, validation, mental health, stress, loneliness

## Abstract

**Background:**

Pet-related stress refers to the stress of living with a pet. The aim of this study was to translate and validate the German version of the Pet-Related Stress Scale (PRSS-G). Moreover, reference values were determined.

**Methods:**

Data for validation were gathered from a quota-based online sample of Germany’s adult population aged 18 to 74 years, with *n* = 3,270 representing the demographic distribution of Germany in terms of sex, age, and federal states. The data collection took place online in January 2025. Reliability was assessed, and confirmatory factor analysis was performed to evaluate construct validity. Concurrent validity was examined through pairwise correlations of PRSS-G with depressive symptoms, anxiety symptoms, perceived stress, life satisfaction and loneliness. Additionally, reference values were provided for key sociodemographic groups.

**Results:**

Strong to excellent reliability was found for the PRSS-G, with Cronbach’s alpha of 0.96 overall and coefficients from 0.88 to 0.96 for the subscales. The mean pet-related stress score equaled 1.9 (SD: 0.8), with the highest levels among younger individuals, individuals with low education and individuals with a migration background. The original three-factor model (economic, psychological and social stress subscales) was confirmed in the present study. Higher pet-related stress was associated with more depressive symptoms (*r* = 0.50, *p* < 0.001), more anxiety symptoms (*r* = 0.48, *p* < 0.001), more perceived stress (*r* = 0.35, *p* < 0.001), lower life satisfaction (*r* = −0.13, *p* < 0.001) and higher loneliness (*r* = 0.30, *p* < 0.001).

**Conclusion:**

The PRSS-G is a reliable and valid tool to measure pet-related stress levels among individuals speaking German. To facilitate comparisons across different countries, additional translation and validation studies are required.

## 1 Introduction

Many people keep pets. This has been the case in the past, but the number of people with pets has significantly increased since the Covid-19 pandemic. For example, in total, 45 percent of all German households had one or more pets in 2023 (1). Moreover, 34.3 million pets lived in German households. The majority of these were cats (15.7 million) and dogs (10.5 million) (1). Overall, 67% of all families with children have a pet in Germany (1). The probability of owning a pet also increases with age in Germany (1).

Studies have shown that interactions with animals or pet ownership is associated with favorable outcomes such as lower loneliness (2) or higher well-being (3). This is attributed, among other things, to the fact that animals can enrich one’s own life, that individuals with animals (e.g., dogs) engage in increased physical activity (4), and have more frequent contact with other individuals who also own pets (5). On the other side, living with a pet can also be associated with unfavorable outcomes. Herzog called this the “pet effect paradox” (6, 7). For example, a recent study showed that cat ownership was associated with higher loneliness levels longitudinally based on community-dwelling individuals aged 40 years and over in Germany (8). This could be because living with a pet can also be associated with certain concerns. For example, individuals living with a pet may be concerned about less time for meeting with friends and acquaintances (9). Additionally, living with a pet can be financially stressful, for example because of pet food, toys for the pet or if the pet becomes ill (10, 11). Moreover, individuals living with pets may be concerned about the well-being (e.g., whether they provide a comfortable environment for the animal) and the health of their pets (e.g., if they become ill or if the animal runs away), but also of their own health - then the question could arise as to who would take care of the pet (11, 12). Thus far, studies dealing with such stress based on validated tools are almost completely lacking. However, this potential stress associated with living with a pet may help to untie the Gordian knot of the pet effect paradox.

There is currently only one tool for quantifying pet-related stress, the Pet-Related Stress Scale (PRSS), developed and validated by Matijczak et al. (based on a convenience sample of 386 individuals living with pets in the United States) (13). The mean age of this previous study was 39.7 years (SD: 12.5 years). Most of the individuals were cisgender women (75.4%). They found, among other things, good internal consistency (McDonald’s omega = 0.88 for the economic stress subscale; psychological stress subscale, McDonald’s omega = 0.89, social stress subscale, McDonald’s omega = 0.93) and their findings supported divergent and convergent validity. This instrument is currently solely available in English language. The aforementioned economic subscale refers to financial strain of individuals living with a pet (e.g., due to the costs of care items or services required for their pets) (13). The psychological stress subscale refers to the emotional strain associated with worries about the well-being and health of pets, one’s own well-being, and pet behavioral issues (13). The social stress subscale refers to pet-related challenges in social relationships and the negative effect of pets on the participation of the individuals in social activities.

Translations and validations of the PRSS in other languages, including German are completely lacking. Consequently, the aim of the current study was translating and validating a German version of the PRSS. This knowledge is important because it allows for future comparisons between countries and enhances our understanding of pet-related stress in the German population. It also helps us gain a better future understanding of the antecedents and consequences of pet-related stress amongst German-speaking communities.

## 2 Materials and methods

### 2.1 Translation process

Our translation process adhered closely to established guidelines (14). A well-known internationally operating translation agency (Tolingo; ISO certified) was responsible for translating the PRSS. Both translators from the agency were native German speakers. One of these had prior experience in the research field, whereas the second one was a “naive” translator (i.e., without background knowledge in this research area). Each translator independently translated the PRSS into German. After that, two German-speaking authors (AH and HHK) harmonized the two versions, with assistance from psychologist (AN). Following this, the harmonized German version was back-translated into English by two native English speakers from Tolingo (again, one naive and one with prior knowledge in this field), both of whom worked independently. Any discrepancies between the two translations were resolved by discussion between AH, AN, and HHK. The final 21-item German version of the PRSS (PRSS-G) is shown in Table 1. Response categories are 1 (never), 2 (a little), 3 (sometimes), 4 (most of the time), and 5 (all the time). By averaging all 21 items (which were all scored in the same direction), a total score was calculated. This score ranges from 1 to 5, with higher scores indicating a higher level of pet-related stress.

**Table 1:** PRSS-G (German version of the Pet-Related-Stress-Scale).

Bitte denken Sie bei der Beantwortung der folgenden Fragen an das Haustier bzw. die Haustiere, mit dem bzw. denen Sie derzeit zusammenleben. Lesen Sie jede Aussage unten und wählen Sie die Option, die am besten beschreibt wie sehr Sie die jeweilige Situation in Bezug auf Ihr Haustier in den letzten 30 Tagen beunruhigt hat. Bitte beachten Sie: Zwecks Lesbarkeit verwenden wir jeweils den Singular (das Haustier). Wir meinen aber jeweils alle Haustiere, mit denen Sie derzeit zusammenleben.					
	Nie	Ein wenig	Manchmal	Die meiste Zeit	Die ganze Zeit
Ich mache mir Sorgen, ob ich mir die Gebühren zum Abholen meines Haustieres aus einem Tierheim leisten könnte, wenn es wegläuft.					
Ich mache mir Sorgen, ob ich meinem Haustier Futter kaufen kann.					
Ich mache mir Sorgen, was mit meinem Haustier passieren würde, wenn ich sterben sollte.					
Ich mache mir Sorgen, dass mein Haustier mich von Dingen abhält, die ich gerne tun möchte.					
Ich mache mir Sorgen, wegen meines Haustieres nicht an sozialen Aktivitäten teilnehmen zu können.					
Ich mache mir Sorgen, ob ich mir Wohnkosten im Zusammenhang mit meinem Haustier (z. B. höhere Miete bzw. Kaution wegen des Haustieres) leisten kann.					
Ich mache mir Sorgen, dass ich notwendige Dienstleistungen für mein Haustier nicht bezahlen kann (z. B. Tierfriseur, Verhaltenstraining).					
Ich mache mir Sorgen, dass mein Haustier wegläuft.					
Ich mache mir Sorgen, dass mein Haustier mein Privatleben einschränkt.					
Ich mache mir Sorgen um die Bezahlung von Tierarztbesuchen für mein Haustier.					
Ich mache mir Sorgen, dass mein Haustier krank werden könnte.					
Ich mache mir Sorgen, dass ich Einladungen ablehnen muss, um bei meinem Haustier zu Hause zu bleiben.					
Ich mache mir Sorgen, dass ich mir Dinge, die mein Haustier glücklich machen, nicht leisten kann (z. B. Spielzeug, Leckerlis).					
Ich mache mir Sorgen, dass ich wegen meines Haustieres nicht ausgehen kann.					
Ich mache mir Sorgen, dass mein Haustier verletzt werden könnte (z. B. von einem Tier, Menschen oder Auto).					
Ich mache mir Sorgen, soziale Veranstaltungen früher verlassen zu müssen, um zu meinem Haustier nach Hause zu gehen.					
Ich mache mir Sorgen, dass mein Haustier mich davon abhält, Zeit mit Freunden zu verbringen.					
Ich mache mir Sorgen, dass mein Haustier nicht glücklich ist.					
Ich mache mir Sorgen, dass mein Haustier sterben könnte.					
Ich habe kein Haustier (_)					

The first statement relates to item 1, while the second statement relates to item 2 etc.; If the option “Ich habe kein Haustier” is selected, then the 19 items must not be answered.

### 2.2 Sample

In our current study, we used data from a large online sample of individuals across Germany, selected based on a quota approach (crossed quota: sex x age; uncrossed: federal state). In total, 3,270 individuals aged 18 to 74 years filled out the questionnaire. Data collection occurred online in January 2025. The market research firm Bilendi, which is ISO certified, conducted the data collection for our present study. A quota-based approach was used to ensure that the sample accurately represented the German adult population in terms of age, sex, and federal state. In total, *n* = 1,923 individuals (out of the 3,270 individuals) had at least one pet in their home. Since the PRSS exclusively focuses on individuals with pets, we focused on such aforementioned individuals in this study.

All participants provided informed consent prior to taking part in our study. Our study received ethical approval from the Local Psychological Ethics Committee, University Medical Center Hamburg-Eppendorf (LPEK-0849).

### 2.3 Other measures

Similar to Matijczak et al. (13), pairwise correlations of the PRSS-G with depressive symptoms, anxiety symptoms, perceived stress, life satisfaction and loneliness were calculated. The established Patient Health Questionnaire-9 (PHQ-9) was used to assess depressive symptoms (15) (Cronbach’s alpha = 0.90, McDonald’s omega = 0.90). By summing up all nine items, a final score was built. This score ranges from 0 to 27 (higher values reflect more depressive symptoms). The well-known Generalized Anxiety Disorder-7 (GAD-7) (16) was applied to measure anxiety symptoms, consisting of seven items. The resulting sum score ranges from 0 to 21, whereby higher values correspond to more anxiety symptoms (Cronbach’s alpha = 0.92, McDonald’s omega = 0.92). Perceived stress was measured using the Perceived Stress Scale (10-item version) (17) (Cronbach’s alpha = 0.84, McDonald’s omega = 0.83). The sum score ranges from 10 to 50, whereby higher scores reflect a higher level of perceived stress. The established Satisfaction with Life Scale (SWLS) developed by Diener et al. (18) was used to quantify life satisfaction. It has five items and the resulting sum score ranges from 5 to 35 (whereby higher values reflect higher levels of life satisfaction; Cronbach’s alpha = 0.91, McDonald’s omega = 0.91). Loneliness was measured using the well-known De Jong Gierveld tool (6-item version) (19). It ranges from 0 to 6, with higher values corresponding to higher loneliness levels (Cronbach’s alpha = 0.77, McDonald’s omega = 0.75). Of note that all of these measures were available in validated German versions.

### 2.4 Statistics

A confirmatory factor analysis (CFA, with ML estimation) was conducted to explore the underlying three-factor structure of the PRSS-G. The following fit indices were computed: Chi-square statistic, Root Mean Square Error of Approximation (RMSEA), Standardized Root Mean Square Residual (SRMR), Normed Fit Index (NFI), Relative Fit Index (RNI), Comparative Fit Index (CFI), and Incremental Fit Index (IFI). To handle non-normality, a Satorra-Bentler adjustment was done (20). Our study adhered to recommendations for establishing good measurement characteristics (21).

To evaluate concurrent validity, Pearson correlations of PRSS-G with depressive symptoms, anxiety symptoms, perceived stress, life satisfaction, and loneliness were calculated and categorized as follows (22): 0.9 or higher: exceptionally high, 0.7 to 0.9: high, 0.5 to 0.7: moderate, 0.3 to 0.5: low, below 0.3: negligible.

To evaluate the reliability of the PRSS-G, Cronbach’s alpha and McDonald’s omega were used. A satisfactory internal consistency was indicated by Cronbach’s alpha values of 0.7 or higher. A strong internal consistency was defined by values of 0.8 or higher and values of 0.9 or above reflect excellent internal consistency (23). There were no missing values in the dataset. Consequently, missing data techniques were not required. Data analysis was performed using StataNow 18.5 (MP-Parallel Edition, Stata Corp., College Station, Texas). We used the user-written Stata commands “validscale” (24) and also “omegacoef” (25). The “validscale” command includes the statistical procedures required to validate tools, while “omegacoef” is used to compute McDonald’s omega in Stata.

## 3 Results

### 3.1 Characteristics of the sample and reference values

The individuals living with at least one pet (*n* = 1,923) had an average age of 44.8 years (SD: 14.9 years); age ranged from 18 to 74 years. Among such individuals, 51.3% were female. The mean pet-related stress score (based on the PRSS-G) equaled 1.9 (SD: 0.8). Sociodemographic groups significantly differed in terms of pet-related stress score. For instance, individuals aged 18 to 29 years had a mean pet-related stress score of 2.4 (SD: 1.0), whereas individuals aged 60 years and older had a mean score of 1.6 (SD: 0.6). This is a large difference (in terms of absolute Cohen’s d of 1.0). Moreover, individuals without a migration background had a mean pet-related stress score of 1.9 (SD: 0.8). In contrast, individuals with a migration background had a mean pet-related stress score of 2.3 (SD: 1.0; absolute Cohen’s d of 0.5). Additional details are shown in Table 2. A detailed description of the items is given in Appendix 1.

**Table 2:** Reference values for pet-related stress (also stratified by key sociodemographic factors).

Variables	*N* (%)	Pet-related stress: mean (SD)	*p*-value
Total sample	1,923 (100.0)	1.9 (0.8)	
Sex			<0.001
Male	934 (48.6)	2.0 (1.0)	
Female	986 (51.3)	1.8 (0.7)	
Diverse	3 (0.2)	2.2 (0.8)	
Age group			<0.001
18 to 29 years	404 (21.0)	2.4 (1.0)	
30 to 39 years	388 (20.2)	2.1 (0.9)	
40 to 49 years	338 (17.6)	1.8 (0.7)	
50 to 59 years	414 (21.5)	1.7 (0.7)	
60 years and older	379 (19.7)	1.6 (0.6)	
Marital status			<0.001
Single	503 (26.2)	2.1 (0.9)	
Divorced	130 (6.8)	1.9 (0.7)	
Widowed	46 (2.4)	1.9 (0.8)	
Living together: Married/Partnership	1,181 (61.4)	1.8 (0.8)	
Living separated: Married/Partnership	63 (3.3)	1.7 (0.7)	
Education			<0.001
Low	220 (11.4)	2.2 (1.0)	
Medium	889 (46.2)	1.8 (0.8)	
High	814 (42.3)	1.9 (0.9)	
Migration background			<0.001
No	1,678 (87.3)	1.9 (0.8)	
Yes	245 (12.7)	2.3 (1.0)	
Employment status			<0.001
Full-time employed	1,025 (53.3)	2.0 (0.9)	
Retired	297 (15.4)	1.6 (0.7)	
Other	601 (31.3)	1.9 (0.8)	

Independent t-tests or oneway ANOVAs were calculated, as appropriate.

For the economic stress subscale, mean score was 1.8 (SD: 1.0). For the psychological stress subscale, mean score was 2.3 (SD: 0.9) and for the social stress subscale, mean score was 1.7 (SD: 0.9). The mean scores of the subscales were significantly different from each other (*p* < 0.001 for each). There were small differences (absolute Cohen’s d = 0.1) between the economic and social stress subscales. Moreover, there were medium to large differences between the economic and psychological stress subscales (absolute Cohen’s d = 0.5) and between the psychological and social stress subscales (absolute Cohen’s d = 0.7).

### 3.2 Reliability

The internal consistency (Cronbach’s alpha and McDonald’s omega) of each PRSS-G factor is displayed in Table 3. The internal consistency was 0.96 for the PRSS-G (Cronbach’s alpha and McDonald’s omega). Both, Cronbach’s alpha and McDonald’s omega also did not differ for all three subscales, i.e., 0.93 for the economic stress subscale, 0.88 for the psychological stress subscale, and 0.96 for the social stress subscale.

**Table 3:** Internal consistency (PRSS-G and subscales).

Internal consistency	PRSS-G	Economic stress subscale	Psychological stress subscale	Social stress subscale
Cronbach’s alpha	0.96	0.93	0.88	0.96
McDonald’s omega	0.96	0.93	0.88	0.96

### 3.3 Validity

#### 3.3.1 Construct validity of the PRSS-G

The three-factor solution which was suggested by Matijczak et al. (13) has been endorsed in this study by means of a CFI. A mostly good model fit (e.g., SRMR = 0.054, CFI = 0.949) was demonstrated. Additional model and fit statistics for the CFA are provided in Table 4. The results of a one-factor solution are shown in Appendix 2.

**Table 4:** Confirmatory factor analysis of data (three-factor solution).

Chi^2^	df	RMSEA	SRMR	NFI	RNI	CFI	IFI
1122.79, *p* < 0.001	149	0.058	0.054	0.941	0.949	0.949	0.949

Satorra-Bentler adjusted goodness-of-fit indices were calculated.

Table 5 depicted the standardized factor loadings for the three-factor solution as well as the Pearson correlations among the three subscales. It is worth noting that the standardized factor loadings mainly ranged from about 0.8 to 0.9 for both the economic stress and the social stress subscale. For the psychological stress subscale, the standardized factor loadings mainly varied from approximately 0.7 to 0.8. The associations between subscales were moderate to high. Further details are presented in Table 5.

**Table 5:** Standardized factor loadings (PRSS-G; SE in parentheses) and Pearson correlations between the subscales.

Items	Economic stress subscale	Psychological stress subscale	Social stress subscale
Item 1	0.81 (0.01)		
Item 2	0.87 (0.01)		
Item 6	0.86 (0.01)		
Item 7	0.86 (0.01)		
Item 10	0.71 (0.01)		
Item 13	0.87 (0.01)		
Item 3		0.71 (0.01)	
Item 8		0.73 (0.01)	
Item 11		0.78 (0.01)	
Item 15		0.77 (0.01)	
Item 18		0.74 (0.01)	
Item 19		0.76 (0.01)	
Item 4			0.83 (0.01)
Item 5			0.88 (0.01)
Item 9			0.86 (0.01)
Item 12			0.89 (0.01)
Item 14			0.91 (0.01)
Item 16			0.88 (0.01)
Item 17			0.88 (0.01)
Economic subscale	1		
Psychological subscale	0.70	1	
Social subscale	0.78	0.61	1

All items were highly significant (*p* < 0.001).

#### 3.3.2 Concurrent validity

Pairwise correlations of the PRSS-G with depressive symptoms, anxiety symptoms, perceived stress, life satisfaction and loneliness are shown in Table 6. A higher pet-related stress score was associated with more depressive symptoms (*r* = 0.50, *p* < 0.001), more anxiety symptoms (*r* = 0.48, *p* < 0.001), more perceived stress (*r* = 0.35, *p* < 0.001), lower life satisfaction (*r* = −0.13, *p* < 0.001) and higher loneliness (*r* = 0.30, *p* < 0.001). Additional details are displayed in Table 6.

**Table 6:** Concurrent validity analysis (with *n* = 1,923 individuals).

Tools	1	2	3	4	5	6
1: German version of the pet-related stress scale (PRSS-G)	1					
2: Depressive symptoms (PHQ-9)	0.50***	1				
3: Anxiety symptoms (GAD-7)	0.48***	0.81***	1			
4: Perceived stress (PSS-10)	0.35***	0.69***	0.72***	1		
5: Life satisfaction (SWLS)	−0.13***	−0.45***	−0.43***	−0.52***	1	
6: Loneliness (DJG-6)	0.30***	0.45***	0.44***	0.46***	−0.49***	1

Pearson correlations are displayed, ^*^p < 0.05, ^**^p < 0.01, ^***^p < 0.001 (bonferroni-adjusted significance levels).

## 4 Discussion

Our present study aimed to translate and validate the German version of the PRSS (PRSS-G) enabling its use in upcoming research amongst German speaking individuals. As the first study, reference values for the PRSS-G were also provided. This enhances our understanding in this research field.

Our present study confirmed the three-dimensional structure suggested by Matijczak et al. (13). This previous study (13) provided adequate fit for such model (three-factor model, RMSEA: 0.063, SRMR: 0.059, CFI: 0.942, TLI: 0.931; using a maximum likelihood estimator with robust standard errors). In our study, we found significant associations of higher pet-related stress with unfavorable mental health, more perceived stress, higher loneliness levels, and lower life satisfaction. Somewhat similarly, Matijczak et al. (13) also found associations between psychological stress (i.e., symptoms of depression, anxiety and somatization) and the three subscales of pet-related stress. Moreover, in our study, strong to excellent internal consistency was shown. Such values were slightly higher than in the original study (13).

Overall, the mean value of the PRSS-G was rather low in our present study. However, certain socio-demographic groups (e.g., people aged 18–29) showed comparatively high values. Thus, future studies should pay special attention to groups that may be disproportionately impacted by economic, psychological, and economic pet-related stress. Because this is the first study to provide reference values for the PRSS-G, we cannot compare such values with results from previous studies.

Further efforts to validate the PRSS-G and original English version may pave the way for comparisons across countries and enrich our understanding of pet-related stress levels and how domains of pet-related stress differ within and across cultures, countries, and identities. Future research based on representative samples of several countries are needed. The strong psychometric characteristics of the PRSS and PRSS-G should encourage other researchers to implement this timely and needed scale in their studies; in particular, assessment of pet-related stress may help to account for unmeasured variability in experiences of pet ownership that lead to inconsistent findings across human-animal interaction studies. Upcoming longitudinal studies could, for example, also explore consequences of pet-related stress in German-speaking populations.

We would like to note some strengths and limitations of our current study: Data were taken from a quota-based sample that is representative in terms of age, sex, and federal states for the general adult population in Germany. A thorough translation process was done in accordance with well-known recommendations (14). This study is the first validation of the German version of the PRSS. Our study had a cross-sectional design, preventing us from computing test–retest reliability and responsiveness.

In conclusion, the PRSS-G is a reliable and valid instrument for measuring pet-related stress among German-speaking individuals. Upcoming validation studies in other languages are required to facilitate cross-country comparisons. Additionally, test–retest reliability should be computed in future longitudinal studies. Upcoming research should also investigate the factors leading to pet-related stress and the consequences of pet-related stress within the German-speaking population.

## Data Availability

The datasets presented in this article are not readily available because data are not publicly available but interested parties may contact the authors for more information. The data are not publicly available due to ethical restrictions. Requests to access the datasets should be directed to André Hajek, a.hajek@uke.de.
