# Supplementary material for: Translation and validation of the German version of the Pet-Related Stress Scale
Source: Front Vet Sci. 2025 May 9;12:1592569. doi: 10.3389/fvets.2025.1592569 (PMC12100280; doi:10.3389/fvets.2025.1592569)
Supplement: Supplementary file 1 [file Table_1.DOCX]

Appendix 1. Item-description

Missing N Response categories Alpha Loevinger Number of

data rate 1 2 3 4 5 - item Hj coeff NS Hjk

-------------------------------------------------------------------------------------------------

Item 1 0.00% 1923 63.03% 13.47% 13.68% 7.54% 2.29% 0.92 0.71 0

Item 2 0.00% 1923 66.72% 11.34% 13.83% 5.62% 2.50% 0.91 0.75 0

Item 6 0.00% 1923 64.07% 12.32% 12.79% 7.80% 3.02% 0.91 0.73 0

Item 7 0.00% 1923 57.72% 15.18% 15.76% 7.75% 3.59% 0.91 0.76 0

Item 10 0.00% 1923 41.50% 19.86% 21.16% 11.02% 6.45% 0.93 0.67 0

Item 13 0.00% 1923 64.90% 13.99% 11.96% 6.19% 2.96% 0.91 0.74 0

-------------------------------------------------------------------------------------------------

Item 3 0.00% 1923 36.61% 22.41% 25.22% 10.19% 5.56% 0.87 0.56 0

Item 8 0.00% 1923 46.54% 22.15% 19.76% 7.64% 3.90% 0.87 0.58 0

Item 11 0.00% 1923 20.02% 21.94% 36.77% 12.64% 8.63% 0.86 0.64 0

Item 15 0.00% 1923 32.71% 23.87% 29.90% 8.27% 5.25% 0.86 0.62 0

Item 18 0.00% 1923 46.44% 21.06% 21.63% 7.90% 2.96% 0.87 0.58 0

Item 19 0.00% 1923 24.13% 21.84% 33.75% 12.58% 7.70% 0.86 0.63 0

-------------------------------------------------------------------------------------------------

Item 4 0.00% 1923 56.68% 18.62% 16.07% 5.98% 2.65% 0.95 0.77 0

Item 5 0.00% 1923 62.56% 15.81% 13.73% 5.72% 2.18% 0.95 0.79 0

Item 9 0.00% 1923 61.83% 17.78% 12.53% 5.82% 2.03% 0.95 0.77 0

Item 12 0.00% 1923 61.99% 15.96% 13.05% 6.71% 2.29% 0.95 0.79 0

Item 14 0.00% 1923 65.00% 14.61% 12.27% 5.98% 2.13% 0.95 0.81 0

Item 16 0.00% 1923 64.01% 15.86% 12.22% 5.93% 1.98% 0.95 0.78 0

Item 17 0.00% 1923 68.90% 12.48% 10.92% 5.51% 2.18% 0.95 0.80 0

-------------------------------------------------------------------------------------------------

Appendix 2. Confirmatory factor analysis (one-factor solution)

| Chi² | df | RMSEA | SRMR | NFI | RNI | CFI | IFI |
| --- | --- | --- | --- | --- | --- | --- | --- |
| 3766.17 p<.001 | 152 | 0.111 | 0.089 | 0.804 | 0.810 | 0.810 | 0.810 |

Notes: Satorra-Bentler adjusted goodness-of-fit indices were calculated.
